# Changes in Surface Tension of Aqueous Humor in Anterior Segment Ocular Pathologies

**DOI:** 10.3390/vision1010006

**Published:** 2016-09-20

**Authors:** Javier Cabrerizo, J. Haritz Urcola, Elena Vecino

**Affiliations:** 1 Department of Ophthalmology, Rigshospitalet/Glostrup, University of Copenhagen, Nordre Ringvej 57, 2600 Glostrup, Denmark; 2 Department of Ophthalmology, University Hospital of Alava, 01009 Vitoria-Gasteiz, Spain; 3 Experimental Ophthalmo-Biology Group (GOBE), University of the Basque Country (UPV/EHU), 48940 Leioa, Spain

**Keywords:** surface tension, aqueous humor, Fuchs endothelial dystrophy, open angle glaucoma, biophysical properties

## Abstract

The aim of this study was to identify and determine differences in surface tension (ST) of aqueous humor (AH) in patients with cataract, glaucoma and Fuchs endothelial dystrophy (FED). Two hundred and two samples of AH were analyzed (control *n* = 22; cataract *n* = 56; glaucoma *n* = 81; and *n* = FED 43). Patients with previous history of anterior segment surgery, anterior segment pathology or intraocular injections were excluded from the study. Different types of glaucoma were identified, cataracts were graded using total phaco time data during surgery and clinical severity of FED was assessed by clinical examination. Around 150 microliters AH were obtained during the first step of a surgical procedure, lensectomy, phacoemulsification, nonpenetrating deep sclerotomy (NPDE) and Descemet membrane endothelial keratoplasty (DMEK). A pendant drop-based optical goniometer OCA-15 (Dataphysics, Filderstadt, Germany) was used to measure surface tension. Mean ST was 65.74 ± 3.76 mN/m, 63.59 ± 5.50 mN/m, 64.35 ± 6.99 mN/m, and 60.89 ± 3.73 mN/m in control, cataract, glaucoma and FED patients respectively. Statistically significant differences between FED and control group were found (*p* < 0.001). Lens condition, cataract maturity, age, and gender did not show influence in ST. ST of AH is significantly decreased in FED patients independently from age and lens condition. These findings may aid to the understanding of the physiopathology of the disease.

## 1. Introduction

Surface tension (ST) is the elastic tendency of fluids caused by intermolecular attraction forces in the surface layer, which makes them acquire the least surface area possible [1]. ST defines the interfacial properties of a fluid [2]. ST of organic fluids is highly dynamic and closely related to the concentration and the type of surface active compounds. These surfactants, mainly proteins and lipids, are characterised by a high surface protein adsorption activity at low bulk concentrations, drastically affecting fluid interfacial properties. As ST is highly sensitive to even small changes in the type and/or concentration of surfactant constituents, it bears diagnostic potential to detect changes in the lipid and protein profile of body fluids, and their changes in pathological conditions [3].

Many surfactants play a significant role in vital functions of the human organism. Surfactant dependency increased in ST, induces narrowing of alveolar capillaries and oxygen desaturation; this being of crucial clinical relevance in neonatology [4].

Increasing evidence suggests the influence of ST in embryological organ development of the vascular system, remodelling vascular patterns and controlling cell to cell adhesion [5]. There are known physiological differences in ST of human fluids related to age, gender and pregnancy, linked with physiological variations of protein concentration in serum and urine [6]. Thus, an inverse correlation exists between ST of serum and concentration of surfactants in healthy subjects. Dovetailing with this, increase of phospholipids in human fluids has been related with a decrease in their ST, showing potential influence in ST regulated physiological processes.

In addition, numerous reports show disease-linked variations in levels of specific surface active molecules in organic fluids [7]. Although in many cases those changes are known to have great influence in the onset of the condition and result from disease progression, in others, its potential etiological relevance remains unknown [8].

Aqueous humor (AH) production is a complex, highly regulated mechanism, with some unknown aspects still to be elucidated. Production and composition of AH is altered under several circumstances: circadian day-night fluctuations [9], systemic conditions [10,11], topical medication [12] and ocular diseases [13].

Proteomic and lipidomic studies of AH suggest an increase in proteins and lipid compounds in some anterior segment pathologies [14,15]. Accordingly, resulting changes in AH interfacial properties with potential clinical significance may be expected. Following this line of work, our study aims to address changes in ST of AH in three common anterior segment pathologies: cataract, glaucoma and Fuchs endothelial dystrophy (FED). These three groups of pathologies share structures that are anatomically and metabolically related to the AH.

## 2. Results

### 2.1. Demographics

Demographic data about age, gender, and lens status are presented in Table 1.

### 2.2. Surface Tension between Groups

**Figure 1:**
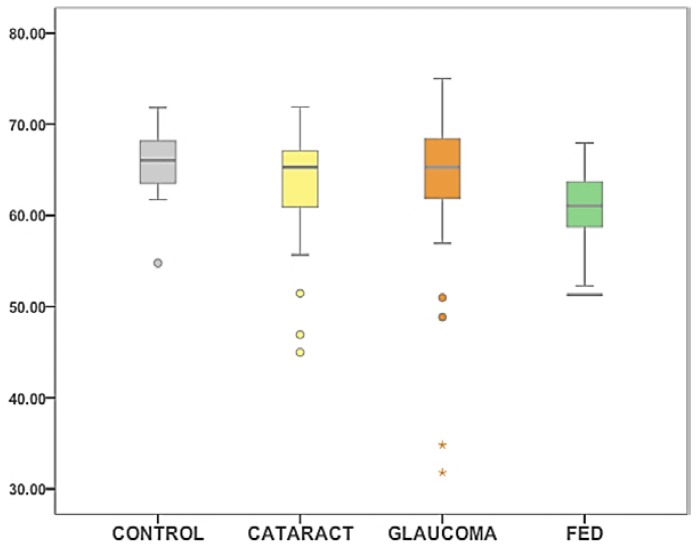
Columns diagram representing ST data for the four groups, controls (grey), cataract (yellow), glaucoma (orange) and FED (green). ST in the *y*-axis, is expressed in mN/m. Outliers are shown as circles (values beyond 1.5 times the interquartile range) and asterisks (values beyond 3 times the interquartile range).

A Mann-Whitney U test was applied to address differences in ST between controls and each group. Statistically significant differences between FED and control group were found (*p* < 0.0001). No statistically significant differences between cataracts and controls and glaucoma and controls were found (Table 2) (Figure 1).

### 2.3. Age/Gender Distribution

The Spearman’s rank test did not find correlation between age and ST and gender and ST.

### 2.4. Lens Condition and Cataract Maturity

**Figure 2:**
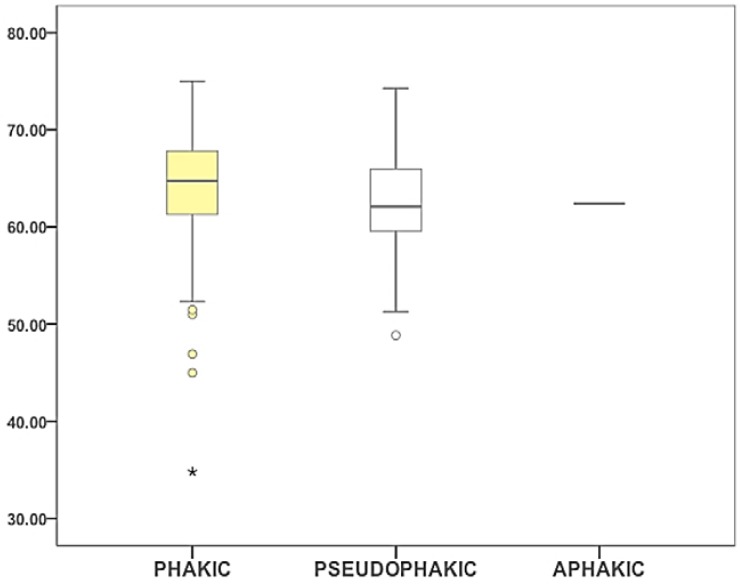
Box-Plot diagram representing ST data for different lens conditions, phakic (yellow), pseudophakic (white) and aphakic (grey). ST in the y-axis is expressed in mN/m. Outliers are shown as circles (values beyond 1.5 times the interquartile range) and asterisks (values beyond 3 times the interquartile range).

We addressed the potential influence of the lens condition (phakic/pseudophakic/aphakic) in ST. No differences in ST between phakic and pseudophakic patients within FED and glaucoma groups were found (*p* = 0.430 and *p* = 0.702 respectively) (Figure 2).

**Figure 3:**
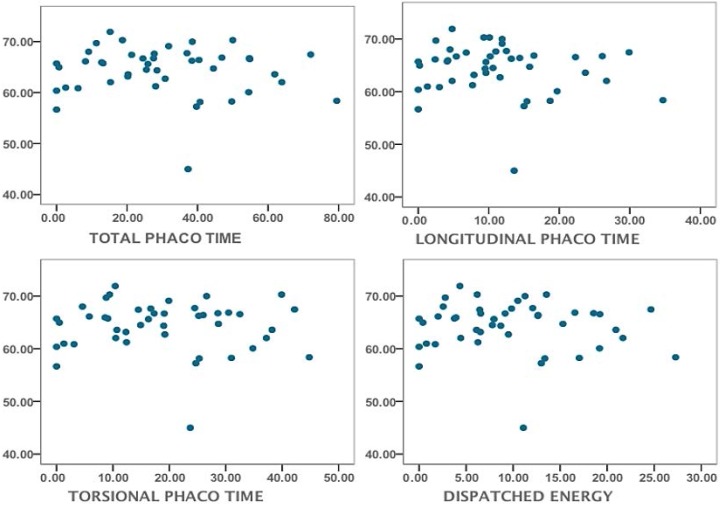
Scatter plots showing the phacoemulsification parameters in 44 cataract cases. Phako time, cumulative and dispatched energy or total phaco energy are expressed in seconds (*x*-axis). ST (*y*-axis) is expressed in N/m. Dispatched energy refers to the total energy delivered into the eye is the product of the phaco power and the absolute phaco time. It is given in seconds of full phaco power.

To study the hypothetical role of cataract maturity in ST we graded cataract cases using the intraoperative phaco parameters. All the surgeries were performed by the same experienced surgeon. Surgery conditions were similar throughout all the cases. No correlation between total phaco time, torsional phaco time, longitudinal phaco time and cumulative dispatched energy and ST was found (Figure 3).

### 2.5. Glaucoma

**Figure 4:**
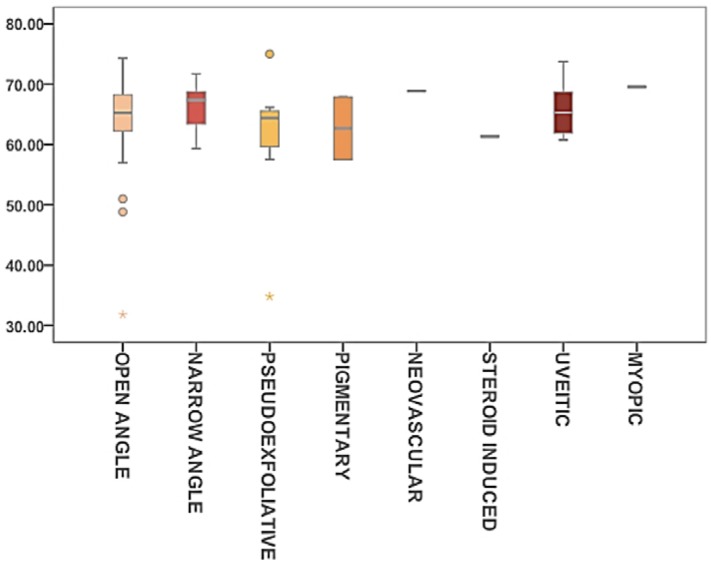
Box-Plot diagram representing ST (mN/m) for different types of glaucoma. Outliers are shown as circles (values beyond 1.5 times the interquartile range) and asterisks (values beyond 3 times the interquartile range).

Eight different types of glaucoma (Table 3) were identified. Kruskal-Wallis one-way analysis of variance was used to address differences between groups. No statistically significant differences in ST data were found between groups (Figure 4).

**Figure 5:**
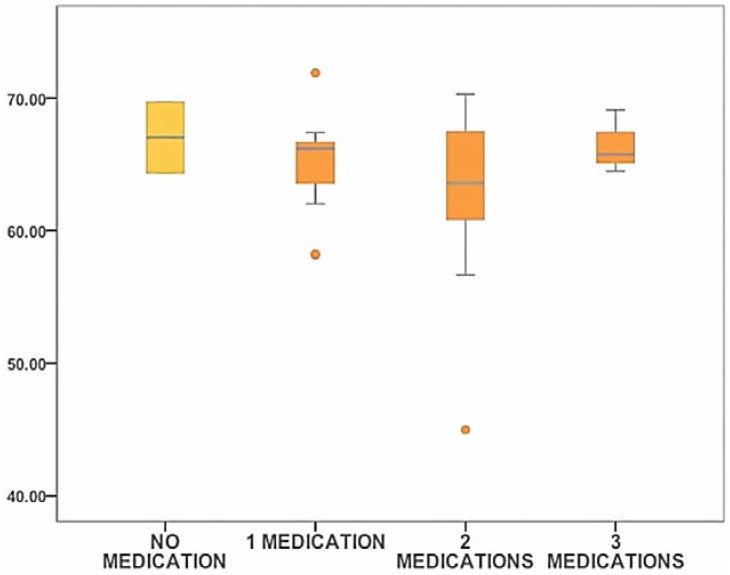
Box-Plot diagram representing ST (mN/m) data for glaucoma cases with different number of daily topical anti-glaucomatous medications. Outliers are shown as circles (values beyond 1.5 times the interquartile range).

The effect of topical medication was also studied. Anti-glaucomatous topical medication within the last six months before surgery was reported. Patients were divided into four groups by the number of medications that they were instilling. No difference was made between one-component medication or fixed-combinations. The number of topical medications did not show an affect on ST data. In addition, no further relations between anti-glaucomatous type and ST were found (Figure 5).

### 2.6. FED

**Figure 6:**
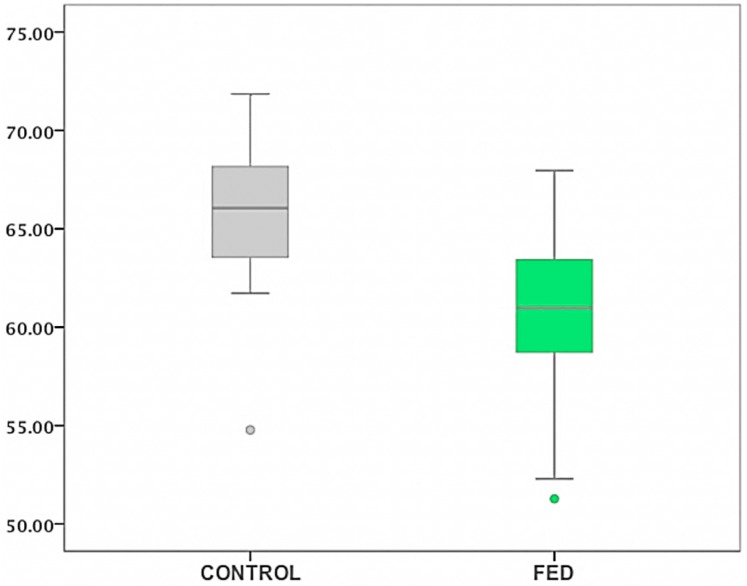
Box-Plot diagram representing ST (mN/m) data for controls and FED. Outliers are shown as circles (values beyond 1.5 times the interquartile range).

Statistically significant differences in ST data were found between FED and controls (*p* < 0.0001) (Figure 6). Kruskal-Wallis rank test was used to address the effect accounted for FED in ST. FED was shown to have 31.4% (*R*^2^ = 0.314) influence in the data of ST. These differences were independent of the lens condition within FED group (*p* = 0.436) (Table 4).

## 3. Discussion

This study unravelled differences in ST between FED AH samples and controls. We did not find differences between age distribution, gender or lens status. These findings suggest disease related variations in concentration of organic surfactants with a potential role in the physiology of the disease.

AH composition in ocular pathologies has been a matter of study in recent years. Differences in AH composition have been associated to common pathologies. Specific changes in lipid peroxidation markers of AH have been found to be a good predictor of cataract maturity [16] and an up-regulated expression of inflammatory proteins has been found in AH of highly myopic patients with cataract. Furthermore, increasing lens capsule permeability in cataract [17] could also affect AH composition. However, lens anterior permeability is reported to be restricted to small and mid-size molecules, with a cut off molecular weight of 166 ± 82 kDa [18], significantly smaller than most organic surfactants. Accordingly, ST did not show to be a good predictor of cataract maturity in our study.

The physiopathology of glaucoma is strongly related to fluid interphase properties at the Schlemm canal and the juxtacanalicular portion of the trabecular meshwork [19]. The main bulk of the outflow resistance takes place at the juxtacanalicular portion [20], where close metabolic relations between epithelial cell and AH also take place potentially altering AH composition [21,22]. In addition, the last part of the transport of AH to the Schlemm canal is a pinocitosis-like process, where giant vacuoles play a role [23]. ST of the fluid influences vacuole forming and stability and may have a potential influence in the final part of AH outflow. Although no statistical differences in ST have been found among the different glaucoma groups, findings have to be taken with caution due to the small number of cases. Future, longer series should help to elucidate expected differences in types of glaucoma where the AH composition is altered, like uveitic or pseudoexfoliative.

Recent studies point to the endothelial cell metabolism as a key to understand the physiopathology of the FED. Findings report an increase in intracellular unfolded protein response [24,25], expression of oxidative stress markers [26], DNA fragmentation [27] down-regulation of protective anti-oxidative stress pathways and subsequent cell apoptosis [28]. Recent works demonstrated changes in the lipidomic profile of AH in FED, mainly related to the increase of lipid compounds, like sphingomyelins and cholesteryl esters, with proven surfactant activity [29]. In addition, AH of FED patients shows changes in the proteomic profile and increase in overall protein levels [30]. Furthermore, the corneal andothelial monolayer and the AH share a relatively large surface area where continuous metabolical interaction takes place. Proteins and lipids, altered in the aqueous humor of FED patients, are expected to influence the ST. Those findings dovetail with our current results, suggesting a relation between the increase of lipids levels and the decrease in ST in AH of patients with FED.

AH is a fast changing, highly dynamic medium and these characteristics constitute the most relevant limitation of our study. Special attention was paid to surgery protocol, topical treatment monitoring, standardization of sample obtention, and storage conditions to minimize spurious data variability. Regulation of the production of AH is highly complex and many of its mechanisms are still only partially known. Thus, this was recognized as one of the potential limitations of our work. We traced for AH production modulators throughout the whole sample processing and no differences regarding day-night fluctuations, oral medication [31] or metabolic conditions [32] were identified. In addition, age distribution could arguably play a role in ST. FED patients are on average around 10 years older than controls and AH composition may change with age. However, multivariate analysis did not show age-related differences in ST.

Patients with FED were under topical fluorometholone 0.1% during 1–2 weeks following laser iridotomy to avoid postoperative air-induced angle closure. Topical steroids alter trabecular meshwork cell morphology by increasing nuclear transport of the human glucocorticoid receptor GRbeta [33] and increase expression and secondary accumulation, of extracellular matrix protein fibronectin, polymerized glycosaminoglycans and elastin [34]. Although reports suggest minor influence of fluorometholone in AH production [35] and most patients ended the treatment days to weeks prior to surgery, potential influence in ST cannot be completely discarded.

Our results contrast with previous reports assessing ST differences in AH of patients with glaucoma where significant differences between both groups were found. Sample size and wider inclusion criteria, including patients that underwent combined glaucoma and cataract surgery, may help to explain those inconsistencies. Due to the challenging aspects of the experimental conditions when using optical goniometry on biological fluids, a strict standardization is key to successfully adapt the field of optical goniometry to clinical research.

## 4. Material and Methods

### 4.1. Design

Two hundred and two samples of AH were analyzed (Table 1). Patients with previous history of anterior segment surgery, anterior segment pathology or intraocular injections were not included in the study. Any topical ocular medications within the last 6 months were reported.

AH in the control group was obtained at the first step of an elective lensectomy or collamer lens ICL implant. All cataract surgeries were performed by the same surgeon. In 44 of them, severity was graded by the following phacoemulsification parameters: total phaco time, longitudinal phaco time, torsional phaco-time and cumulative dispatched energy. Data were generated by the phacoemulsification device (Infinity, Alcon, Fort Worth, TX, USA). Different types of glaucoma were identified and classified in 8 groups as follows: open angle (*n* = 55), narrow angle (*n* = 7), pseudoexfoliative (*n* = 8), pigmentary (*n* = 2), neovascular (*n* = 1), steroid induced (*n* = 1), uveitic (*n* = 6) and myopic (*n* = 1). Diagnosis of FED was performed through clinical slit lamp examination, specular microscopy, and corneal pachimetry. Clinical severity has been graded at the slit lamp by assessing the confluence and area of guttae, and the presence of posterior or full thickness corneal edema [36].

### 4.2. Ethical Statement

The research was approved by the competent institutional human experimentation committee, Comite Ético de Investigación Clínica de Euskadi (CEIC-E)/HUA2012. Following the guidelines of the committee, informed consent was obtained from the subjects prior to the enrollment, after explanation of the nature and purpose of the study. The research reported in this study adhered to the tenets of the Declaration of Helsinki.

### 4.3. Sampling

AH was obtained during the first step of a surgical procedure, refractive lensectomy, phacoemulsification, non perforating deep sclerectomy (NPDE) and Descemet membrane endothelial keratoplasty (DMEK), in controls, cataract, glaucoma and FED patients respectively. After topical 1% povidone-iodine instillation, around 150 microliters of AH were directly aspirated through a unique corneal side port using a 27-gauge blunt cannula. No noteworthy adverse events were noticed during the aspiration process. A moderate shallowing of the anterior chamber was occasionally perceived. AH was directly introduced in a 0.5 mL tube (Eppendorf AG, Hamburg, Germany) after aspiration and stored at −80 °C at the tissue bank for up to 9 months (range: 1–9). Prior to the analyses, samples were left at 24 °C for three hours to gradually adjust to room temperature.

### 4.4. Venue

Control samples were obtained at Begitek Clinica Oftalmologica (Plaza Teresa de Calcuta, 7, 20012 Donostia, Guipúzcoa, Spain). Cataract and glaucoma cases were obtained at Araba University Hospital (C/Jose Atxotegi, s/n, 01009 Vitoria-Gasteiz, Álava, Spain). FED samples were obtained at Netherlands Institute of Innovative Ocular Surgery (Laan op Zuid 88 3071 AA Rotterdam, The Netherlands). Surface tension analysis was performed at the Laboratory of Experimental Ophthalmology at the University Hospital of Alava.

### 4.5. Sample Manipulation and Storage

After obtention, samples were stored frozen at −80 °C at the research tissue bank for up to 9 months (range 1–9). Prior to the start of the analyses, samples were left at 24 °C for three hours to gradually adjust to room temperature.

### 4.6. Surface Tension Analyses

**Figure 7:**
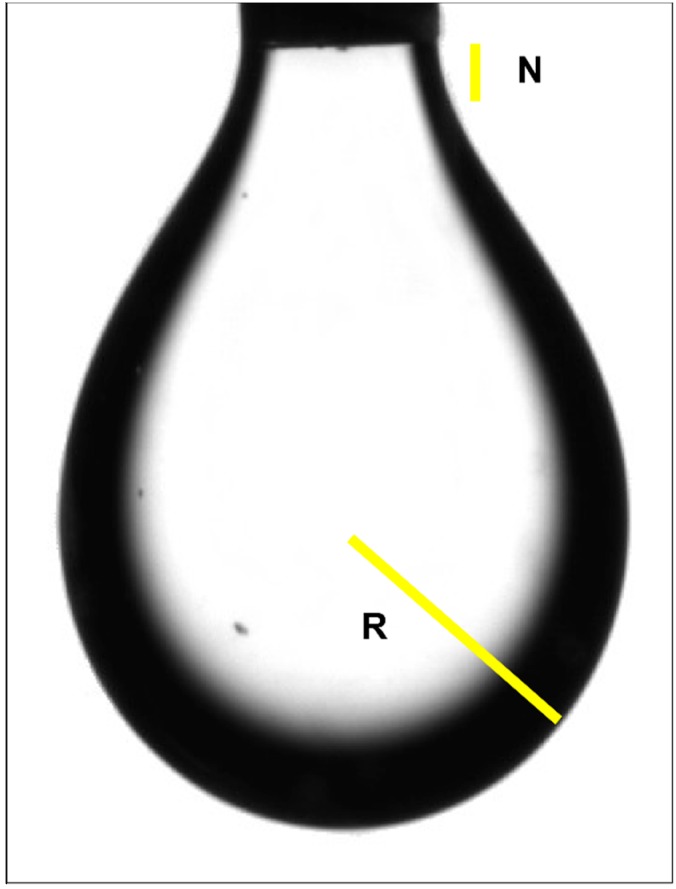
Snapshot of a pendant drop at the moment of ST calculation. The neck area (N) in the superior segment of the rop, defines the critical hydrostatic energy point. Radius of curvature at the apex of the drop (R) is calculated by the software.

A pendant drop method-based optical goniometer, OCA-15 (Dataphysics, Filderstadt, Germany) was used to measure ST. The technique involves the acquisition of a silhouette of an axisymmetric fluid droplet, and the iterative fitting of the Young-Laplace equation that balances gravitational deformation of the drop with the restorative interfacial tension. The unit consists of a computerized optical video camera and a high precision syringe holder. The AH is aspirated by an 18-gauge metal cannula connected to a 200 µLmetal precision syringe (Hamilton, Reno, NV, USA) and is placed vertically in the syringe holder. A software controlled system activates a motor which presses the piston of the syringe at constant speed. When the fluid reaches the tip of the cannula, a drop starts to form. The high quality optical camera takes a snapshot of the drop at the critical hydrostatic energy point (Figure 7).

This point is characterized by a visible “neck” in the transition between the fluid and the tip of the cannula. The radius of curvature at the apex of the drop (R) is calculated. Using the Bashford-Adams method, the ST is inferred by applying the Young-Laplace equation to the outline of the drop. ST results are given in accordance to the International System of Units in Milinewton per meter (mN/m).

Density, altitude and temperature are recognized as crucial parameters to alter surface tension of a fluid. We used bovine aqueous humor density (1.007 g/cm^3^) for calibration, as the closest estimation available in the literature [37]. Room temperature was monitored throughout the whole process and was kept stable at 24 °C. 9 samples (3 control group, 1 cataract group, 5 FED group) were dismissed due to insufficient sample volume and are not included in the demographics of the study [38,39,40].

### 4.7. Data Processing

The data were processed using the device specific software. 40 measurements were taken of every optimal drop image. Only results within a range of 0.2 were considered valid. Higher deviations were considered perturbations on accuracy due to vibrations and measurements were repeated [41]. This threshold was set due to 20 previously repeated measurements with ultrapure water type1 (Mili-Q, Millipore Corporation, Darmstadt, Germany). The SD of the 20 measurements was 0.045. For the ST data, maximal accepted standard error was set at 1.2. Results variability and instrument related measurement bias were studied in a series of 20 repeated measurements with ultrapure water type1 (Mili-Q, Millipore Corporation, Darmstadt, Germany) with very low SD (0.045). SPSS21 (IBM, Armonk, NY, USA) was used for the statistical analysis of the data.

## 5. Conclusions

In summary, our study demonstrates changes in ST of AH in FED, which may be related to variations in protein and lipid composition of AH in FED patients. The relation between ST changes and the concentration of specific, surface active lipids and proteins and their potential role in the pathophysiology of the disease remain future challenges.

## Figures and Tables

**Table 1:** Demographic data displaying age, gender and lens condition within each of the 4 groups.

Demographic Data	Control	Cataract	Glaucoma	FED	Total
Age Average (Range)	52.09	74.4	70.81	70.42	69.69
	(28–80)	(53–102)	(18–92)	(53–87)	(18–102)
Gender Male/Female	8/14	13/43	43/38	21/21	85/116
Lens Condition Phakic/Pseudophakic	22/0	56/0	57/24	11/32	146/56
Number of Cases	22	56	81	43	202

**Table 2:** Descriptive analysis of surface tension (ST) data.

Descriptives of ST Data	Mean	Median	Variance	Standard Deviation	Range
Control	65.74 mN/m	66.05 mN/m	14.26	3.78	17.07
Cataract	63.59 mN/m	65.29 mN/m	30.28	5.50	26.92
Glaucoma	64.35 mN/m	65.29 mN/m	48.88	6.99	43.21
FED	60.89 mN/m	61.16 mN/m	13.89	3.72	16.69
All	63.55 mN/m	64.21 mN/m	34.44	5.87	43.21

**Table 3:** ST statistical analyses between the different glaucoma groups.

ST in Different Types of Glaucoma	*N*	Mean (mN/m)	SD	Mean Rank
Open Angle	55	64.35	6.8	41.0
Narrow Angle	7	66.1	4.4	46.4
Pseudoexfoliative	8	61.13	11.7	33.5
Pigmentary	2	62.7	7.4	31.5
Neovascular	1	68.89	-	65.0
Steroid induced	1	61.31	-	18.0
Uveitic	6	66.38	4.9	43.4
Myopic	1	66.56	-	69.0
Total	81	81	7.0	-

KRUSKAL-WALLIS TEST: Chi-Square 4.988; Asymp. Sig 0.661.

**Table 4:** ST statistical analyses between control and FED and between phakic and pseudophakic FED cases.

ST in FED vs. Controls	*N*	Mean Rank
Controls	22	47.45
FED	43	25.60
Phakic FED	11	19.45
Pseudophakic FED	32	22.88

Asymp. Sig. (Mann-Whitney Test) *p* < 0.001; Sig. (Mann-Whitney Test) *p* = 0.436.
